# Detection of *Mycoplasma* DNA Using Conventional Polymerase Chain Reaction in Canine Abortion, Stillbirth, and Neonatal Mortality Cases in Central Italy

**DOI:** 10.3390/pathogens13110970

**Published:** 2024-11-06

**Authors:** Maria Luisa Marenzoni, Filomena Chierchia, Lakamy Sylla, Elisa Rossi, Michela Beccaglia, Daniele Marini, Iolanda Moretta, Vincenzo Cuteri

**Affiliations:** 1 Department of Veterinary Medicine, University of Perugia, Via San Costanzo 4, 06126 Perugia, PG, Italy; marialuisa.marenzoni@unipg.it (M.L.M.); filomena.chierchia@virgilio.it (F.C.); lakamy.sylla@unipg.it (L.S.); elisarossi37@gmail.com (E.R.); daniele.marini@dottorandi.unipg.it (D.M.); 2 Ambulatorio Beccaglia, Via P.R. Giuliani 32, 20851 Lissone, MB, Italy; ambulatoriobeccaglia@gmail.com; 3 School of Biosciences and Veterinary Medicine, University of Camerino, Via Circonvallazione 93/95, 62024 Matelica, MC, Italy; vincenzo.cuteri@unicam.it

**Keywords:** *Mycoplasma* spp., dog, abortion, neonatal mortality, sequencing

## Abstract

*Mycoplasma* spp. has been involved in canine infertility, but research on this topic is limited and shows conflicting results, as it has also been isolated from vaginal swabs of healthy dogs. This study aimed to retrospectively research *Mycoplasma* DNA by a conventional dual Polymerase Chain Reaction from 114 cases of canine abortion, stillbirth, and neonatal mortality that occurred in Central Italy. In addition, four fetal membranes from dystocic dams were analyzed. In total, 7 out of 114 cases (6.14%) and one of the fetal membranes tested positive for *Mycoplasma* DNA. From five of them (62.5%), other microorganisms were identified, particularly *Canid herpesvirus-1* (2/8, 25%) and *Escherichia coli*, *Staphylococcus aureus*, and/or *Staphylococcus pseudintermedius* (3/8, 37.5%), notoriously responsible for infertility in bitches or neonatal mortality. In two different litters, only one puppy of each one tested positive for *Mycoplasma* DNA. Additionally, *Mycoplasma* DNA was detected in fetal membranes collected during a cesarean section of a bitch whelping *Mycoplasma*-negative puppies, supporting that *Mycoplasma* spp. is part of the normal genital microflora of the female. The detection of *Mycoplasma* DNA in association with other major pathogens and its detection in the female genital tract without transmission to puppies support the hypothesis that *Mycoplasma* belongs to the autochthonous genital microflora or, at most, may play a secondary role in canine abortion and neonatal mortality.

## 1. Introduction

Abortion, stillbirth, and neonatal mortality are significant reproductive issues in canine breeding, with reported incidences ranging from 5% to 35%. The etiology of these reproductive losses is complex and can be broadly categorized into infectious and non-infectious causes [1]. Infectious agents are particularly implicated in the high mortality rates observed during the first week of life. Among the main bacterial pathogens, *Staphylococcus* spp., *Streptococcus* spp., and *Escherichia coli*, *Brucella canis* are frequently identified as primary causes of pregnancy loss and neonatal death in bitches [2,3,4]. Viral infections, including canine herpesvirus (CHV-1), canine parvovirus (CPV), and canine minute virus, are also recognized contributors to these reproductive failures. Additionally, protozoal infections such as *Neospora caninum* may rarely result in abortion and neonatal mortality [5]. However, despite comprehensive diagnostic efforts, a considerable percentage of cases remain of unknown etiology, with 55% of canine neonatal deaths, for instance, being attributed to undetermined causes [6]. These undetermined causes may be linked to hereditary or genetic factors (such as lethal genes), placental insufficiencies, hormonal problems, or rare causes, including infectious ones, which are undiagnosed. A previous study has attempted to attribute rare infectious causes to this subset of undiagnosed cases [7], but the possibility of other atypical or less commonly investigated potential etiological agents, such as *Mycoplasma*, remains.

*Mycoplasma* species have been implicated in reproductive issues in various species, including canines, but their exact role remains controversial due to their frequent isolation from both healthy and affected individuals [8,9,10].

*Mycoplasma* spp. (genus currently named *Mycoplasmopsis*, class Mollicutes, order Mycoplasmatales, family Mycoplasmataceae [11]) have been isolated from the genital tract of both healthy and infertile male and female dogs and are considered part of the normal vaginal flora [12,13,14]. The most common *Mycoplasma* reported in the urogenital tract is *Mycoplasma canis* (currently taxonomically named *Mycoplasmopsis canis*), identified in healthy and infertile dogs, including those with urogenital diseases [8,9,15,16], while *M. edwardii* (currently taxonomically named *Mycoplasmopsis edwardii*) is mostly reported in healthy dogs [10]. Other species identified in the genital tract of the dog include *M. molare* (currently named *Mesomycoplasma molare*), *M. spumans* (currently named *Metamycoplasma spumans*), and *M. maculosum* (currently named *Mycoplasmopsis maculosa*), which have variable but generally lower prevalences than *M. canis* [8,9,10]. Experimental studies have demonstrated the potential role of *M. canis* as a pathogen [12,17,18], and other *Mycoplasma* spp. have been associated with canine infertility [16,19,20] or pyometra [21]. However, the existing studies are very limited and somewhat contradictory. Experimental and observational studies have recognized *Mycoplasma* spp. as a cause of endometritis, abortion, and neonatal mortality in many different species, such as cats, cattle, sheep, goats, and humans [22,23,24,25,26].

Canine mycoplasmas can be considered a model for mycoplasmosis research in the human genital tract due to their taxonomic similarity [20]. Also, canine *Mycoplasma* can colonize human bodies [27,28].

The specialization in *Mycoplasma* isolation and the metabolic needs of these bacteria make their identification more difficult than other bacteria [15]. Culture-independent methods, such as biomolecular methods, have been found to be more sensitive for the identification of *Mycoplasma* species, possibly because the colonization of mucosal tissues is minimal or the viability of the microorganism is often compromised [29].

The purpose of the present study was to retrospectively detect *Mycoplasma* DNA in a caseload of canine abortion, stillbirth, and neonatal mortality submitted to a veterinary microbiology laboratory to identify infectious agents associated with canine abortion.

## 2. Materials and Methods

### 2.1. Sampling

During the period from 2006 to 2019, pools of lung, liver, and/or spleen from fetuses or prematurely dead newborns and, when available, fetal membranes and vaginal swabs of the dam were collected from cases of canine abortion (defined as fetal loss during the second half of pregnancy and characterized by the expulsion of a stillborn or a living incapable of independent life), stillbirth (defined as puppies reported dead at birth), and dead neonatal puppies (defined as puppy death occurred within 28 days of birth) that occurred in Central Italy. The samples were referred to the microbiology laboratory of the Department of Veterinary Medicine of Perugia to be tested for the presence of viral and/or bacterial pathogens. In individual cases, the pool of tissues consisted of other organs (for example, case no. 3, including lung, spleen, and kidney) based on any lesions appreciable at necropsy.

All the data presented in this study were collected during routine diagnostic procedures, and therefore, no specific ethical approval was required. The samples were collected for diagnostic purposes, along with the associated anamnesis. Additionally, the samples were anonymized to ensure privacy and confidentiality.

In this study, a total of 162 specimens were examined, representing 114 cases collected from a minimum of 91 different litters. These included 10 abortions (8.8%), 4 stillbirths (3.5%), 78 cases of neonatal mortality (68.4%), and 22 cases in which the exact age of the newborns was not documented (19.3%). In addition, four fetal membranes from dystocic dams were analyzed. Samples were sent to the laboratory within 24 h of the event (abortion, stillbirth, or neonatal mortality) and were kept at 4 °C until further processing. When the veterinarians were not able to send the samples within 24 h, it was stored frozen and shipped later.

Based on a screening approach that detects the main infectious and non-infectious causes of infertility, abortion, stillbirth, and neonatal mortality, a complete protocol was performed, when possible, including necropsy and histological examinations associated with bacteriological and virological tests. However, economic reasons, method of storage, or delivery of the samples limited the possibility of performing all tests in each case. In particular, bacteriological testing may be compromised, especially for certain bacteria, while molecular tests are less affected by this issue. This is the reason why not all frozen samples were subjected to bacteriological isolation analysis.

The biomolecular method was used to detect the viral agents (*Canid herpesvirus-1* and *Canine parvovirus*), while a standard bacteriological examination (seed onto Blood agar, Mannitol-salt agar, and MacConkey’s agar plates—Oxoid, Milan, Italy), incubated for at least 24 h at 37 °C under aerobic and anaerobic conditions (GasPak^TM^ EZ, Becton Dickinson, Franklin Lakes, NJ, USA), was carried out to detect the main bacterial pathogens. The isolated bacteria were identified by using the MALDI-TOF MS (SOP Direct Transfer Procedure Revision.4; Bruker Microflex Lt^®^, Bruker Daltonics, Bremen, Germany). If more than three bacterial species were isolated from the same sample, they were considered as contaminants.

### 2.2. Biomolecular Investigations

DNA extraction from fetal or neonatal tissues was performed using the GenElute Mammalian Genomic DNA Miniprep Kit (Sigma-Aldrich, St. Louis, MO, USA). The concentration and purity of the extracted DNA were quantified using a NanoDrop spectrophotometer (NanoDrop2000, ThermoFisher Scientific, Milan, Italy). The extracted DNA was stored at −20 °C until use.

A conventional dual Polymerase Chain Reaction (PCR) protocol targeting the 16S rRNA gene was used to detect *Mycoplasma* spp. [30,31]. The primers are listed in Table 1. An in silico simulation, as previously described [32], was conducted to confirm the primer specificity for detecting canine *Mycoplasma* pathogens using Primer-BLAST (https://www.ncbi.nlm.nih.gov/tools/primer-blast/, accessed on 22 June 2021). PCR products of the expected size were purified using a PCR purification kit (Qiaquick, Qiagen, Italy) and directly sequenced on both strands with the same primers as previously described by a DNA analyzer (ABI 3730, Applied Biosystems, Waltham, MA, USA) capillary sequencer (Bio-Fab Research srl, Rome, Italy). Sequences were assembled and aligned using BioEdit (Version 7.2.5, 2022). Sequence similarity was checked against sequences deposited in GenBank using the BLAST 2.13.0+ (NCBI, Bethesda, MD, USA; released 21 February 2022) to confirm the species specificity of the PCR.

## 3. Results

### 3.1. In Silico Analysis

The in silico analysis of the used primers revealed that they were able to simultaneously amplify the DNA of almost all *Mycoplasma* species isolated in dogs. They do not amplify microorganisms previously classified as *Mycoplasma*, such as *M. haemocanis* (currently, class Mollicutes, order Mycoplasmatales, family Mycoplasmataceae, genus *Mycoplasma*), *Acholeplasma* (currently, class Mollicutes, order Acholeplasmatales, family Acholeplasmataceae, genus *Acholeplasma*) and *Ureaplasma* (currently, class Mollicutes, order Mycoplasmoidales, family Mycoplasmoidaceae, genus *Ureaplasma*). Moreover, a significant primer identity with *Bacillus cereus* was detected, highlighting the need to sequence the amplified products to assess the actual identity of the sequences.

### 3.2. Prevalence of Mycoplasma spp. and Other Pathogens in Abortion and Neonatal Mortality Cases

On the basis of the diagnostic protocol above described, although not systematically investigated, probable causes of abortion and neonatal mortality were identified in 49 out of 114 cases, accounting for 47.6%: CaHV-1 in 7.8% of cases (6 out of 77), and bacteria in the 61.3% (38 out of 62 cases), with *E. coli* (20/38, 52.6%), *Staphylococcus* spp. (19/38, 50%), *Pseudomonas* spp. (8/38, 21%), *Proteus* spp. (8/38, 21%), *Klebsiella* spp. (8/38, 21%), *Streptococcus* spp. (1/38, 2.6%), *Enterobacter* spp. (1/38, 2.6%), and *Enterococcus* spp. (1/38, 2.6%). These results are summarized in Table 2.

In total, 7 out of 114 cases (6.14%) and 1 of the analyzed fetal membranes tested positive for *Mycoplasma* DNA. The results of the *Mycoplasma*-positive cases, including other microbiological examinations and sequencing, are summarized in Table 3. In five of them (62.5%), other microorganisms were identified in combination, in particular with CaHV-1 (2/8, 25%) and *E. coli*, *Staphylococcus aureus*, and/or *Staphylococcus pseudintermedius* (3/8, 37.5%). In two different litters (cases nos. 2 and 6), only one pup from each one tested positive for *Mycoplasma* DNA.

Sequencing identified *Mycoplasma* species already detected in dogs in the genital tract, except for one case of unidentified *Mycoplasma* previously identified in free-living birds (accession number: KM485604.1), while another uncultured *Mycoplasma* has already been detected in dogs (accession number: EU772945.1).

*Mycoplasma* DNA was identified from a vaginal swab and fetal membrane collected during the cesarean section of a bitch whelping *Mycoplasma*-negative puppies (case no. 9), but sequencing was repeatedly unsuccessful for the sequence detected from the vaginal swab, making it impossible to study their identity. In case no. 8, the *Mycoplasma* species detected in puppy and vaginal swab were different.

## 4. Discussion

*Mycoplasma* has been reported in the canine genital tract. The prevalence of *Mycoplasma* infection in the genital tract ranges from 17% to approximately 80%, with *M. canis* being the most represented species [9,10,15,16,33]. However, the detection of *Mycoplasma* DNA in cases of abortion, stillbirth, and neonatal mortality in dogs has been rarely investigated [2]. In the present study, out of a total of 114 cases of abortion, stillbirth, and neonatal mortality analyzed, together with four cases of fetal membrane and a vaginal swab of the bitch, only eight cases resulted positive for *Mycoplasma* DNA, revealing that the presence of this pathogen is rare in this kind of samples.

In our caseload, other major pathogens associated with abortion and neonatal mortality were identified in 50%, in agreement with the current literature [2,3,4]; in the remaining cases, it was not possible to perform any bacteriological examination due to the method of storage or because it was not required by the submitting party. It should be remembered that a portion of abortions and perinatal mortality is attributed to non-infective causes, including hereditary genetic causes, lethal genes, placental issues, or may also involve hormonal problems, which constitute a fraction of these deaths [4] and not investigated and reported in the current study. Among infectious agents associated with the presence of *Mycoplasma* DNA, CaHV-1 was detected in cases nos. 2 and 8, while cases nos. 1 and 6 showed *E. coli* with or without *S. pseudintermedius*. These findings are consistent with the existing literature, recognizing these agents as a cause of abortion and neonatal mortality [2,3,4].

*Mycoplasma* DNA was detected only in some dead puppies of a littermate, both in the presence of other main pathogens (such as cases nos. 2 and 6) and not. This suggests that *Mycoplasma* spp. is a normal component of the reproductive tract microflora, not responsible for mortality, at least alone, and other factors should be taken into consideration as potential contributors to the mortality. However, additional research and diagnostic testing could help to better define the role of *Mycoplasma* spp. in these cases (i.e., investigating virulence factors of specific *Mycoplasma* strains, the immune response of the puppies, or any potential interactions between *Mycoplasma* and other pathogens or pathological condition in the reproductive tract).

The presence of *Mycoplasma* in vaginal swabs and fetal membranes from normal pregnancy (case no. 9), which tested positive for *Mycoplasma* (associated with *E. coli* and *S. aureus*), with healthy puppies tested negative after cesarean section, further supports the hypothesis that *Mycoplasma* may be a normal component of the genital tract. On the contrary, case no. 8 initially suggested a potential mother-to-fetus transmission of *Mycoplasma*, as both the puppy and the corresponding vaginal swab from the dam tested positive for *Mycoplasma*. However, sequencing revealed that they were two different *Mycoplasma* species. More extensive studies on the detection of *Mycoplasma* DNA during all canine whelping events, including those categorized as normal, could be useful to determine whether *Mycoplasma* is a part of the normal microbial flora during this specific period in the birth canal.

Sequence typing of these eight *Mycoplasma* spp. identified species allowed to confirm the species already detected in the genital tract by other authors [8,10,20], except for two cases (nos. 4 and 8) in which uncultured *Mycoplasma* was detected. Only a few bacterial species, such as *M. maculatum* and *M. spumans*, have previously been associated with infertility in dogs [16,19,20,33,34]. The identification of pathogenic roles of different *Mycoplasma* species in dogs is limited, and not all species are pathogenic or highly virulent [20]. *M. cynos*, known to be pathogenic in the respiratory system, has also been identified in the genital tract in other studies [8,15,16,35,36,37].

Abnormal detection of *Mycoplasma* DNA in the intestine was observed in one case (no. 4), although 30% of healthy dogs have been reported to have *Mycoplasma* in the colon [38].

The lack of complete anamnestic data and the great heterogeneity of the samples limited the evaluation of cases. Additionally, the retrospective nature of the study made it difficult to interpret the epidemiological framework. Specifically, the absence of bacteriological examinations in some samples, the failure to search for other viruses, and incomplete necropsy and histological investigations in cases positive for *Mycoplasma* prevent conclusive determination of other potential causes of mortality. Considering the overall results, *Mycoplasma* spp. can be considered a normal component of the microflora or possibly an opportunistic pathogen when present with other primary pathogens. To address these limitations and improve the understanding of the role of *Mycoplasma* in these kind of samples, a structured surveillance program encompassing a larger number of cases, along with a standardized form for recording clinical signs and potential risk factors, should be implemented.

The present study obtained only qualitative data (presence/absence of bacterial DNA), but future research should explore quantitative aspects to gain a deeper understanding of the role of *Mycoplasma*, as reproductive tract infections are believed to be caused by overgrowth of normal microflora. Recently, a relative abundance of *Mycoplasma* has been detected in the endometrial microbiome of dogs with pyometra [21]. However, in another study, the number of microorganisms was not considered a relevant factor, while the duration of the infection, the degree of epithelial damage, or the local immune response were hypothesized to be more relevant in the high quality of the semen [39]. In this specific context, further tests such as immunohistochemistry and in situ hybridization are required to determine the role of mycoplasmas in causing abortion or neonatal mortality, similar to what has been observed in other species [24].

## 5. Conclusions

The involvement of *Mycoplasma* in canine abortion and neonatal mortality remains one of the least studied causes, and, in any case, its role seems to be limited. Future investigations should also consider quantitative aspects and examine the specific association of bacteria with the lesions using histological in situ techniques to determine their role in the development of abortion, stillbirth, and neonatal mortality in dogs.

## Figures and Tables

**Table 1:** Sequences and characteristics of the primers used for *Mycoplasma* detection.

Primer	Sequence (5′–3′)	Amplicon Size (bp)	Target	Reference
GPF	GCTGGCTGTGTGCCTAATACA	1013	16S rRNA gene of *Mycoplasma* spp.	Lierz et al., 2007 [31]
MGSO	TGCACCATCTGTCACTCTGTTAACCTC			
MCPL-F	GGGAGCAAACAGGATTAGATACCCT	273	16S rRNA gene of generic *Mycoplasma*	Vojdani et al., 1999 [30]
MCPL-R	TGCACCATCTGTCACTCTGTTAACCTC			

**Table 2:** Results for the pathogen detection and identification in canine abortion and neonatal mortality cases of the present study.

Causes of Abortion and Neonatal Mortality	Number of Cases out of Number of Samples Investigated (Percentage)
**Viral Causes**	
*Canine Herpesvirus 1* (CaHV-1)	6/77 (7.8%)
**Bacterial Causes**	38/62 (61.3%)
*Escherichia coli*	20/38 (52.6%)
*Staphylococcus* spp.	19/38 (50%)
*Pseudomonas* spp.	8/38 (21%)
*Proteus* spp.	8/38 (21%)
*Klebsiella* spp.	8/38 (21%)
*Streptococcus* spp.	1/38 (2.6%)
*Enterobacter* spp.	1/38 (2.6%)
*Enterococcus* spp.	1/38 (2.6%)
*Mycoplasma*-positive cases	8/114 (7%)
***Mycoplasma*** **-positive cases identified in combination with additional microorganisms**	5/8 (62.5%)
Combined with CaHV-1	2/8 (25%)
Combined with *E. coli*, *Staphylococcus aureus*, and/or *Staphylococcus pseudintermedius*	3/8 (37.5%)
Total cases investigated	114 *

* The total number of investigated cases does not reflect the overall number of samples examined reported in the column, as some samples were subjected to both bacteriological and virological testing, while others were only tested for specific pathogens.

**Table 3:** Summary of *Mycoplasma* DNA-positive cases: sample types, sequencing results, virological, and bacteriological examinations for each case.

Case No.	Type of Samples	History	Results of Sequencing *	CaHV-1 PCR Result	Bacteriological Examination
1	Pool of tissues	Puppy, 7 days old, died without microscopic lesions; the entire litter is deceased.	*Mycoplasmopsis canis* (99% with LR215010.1) or *Mycoplasmopsis cynos* (99% with HF559394.1)	-	*Staphylococcus pseudintermedius*, *Escherichia coli*
2	Pool of tissues	Puppy, 15 days old. The entire litter perished at 8–15 days of life. They did not receive colostrum. The dam was vaccinated against CHV-1.	*Mycoplasmopsis canis* (100% with LR215010.1)	+	Not executed
3	Gut and pool of tissues (lung, spleen, and kidney, LSK)	Puppy, 28 days old, died. It comes from a breeding farm where another 60 puppies died due to Canine parvovirus infection.	Gut: *Mycoplasmopsis cynos* (100% with HF559394.1) LSK: *Mycoplasmopsis cynos* (99% with HF559394.1)	-	Not executed
4	Gut	Puppy died at 15 days of life. The other littermates were healthy.	Uncultured bacterium (100% with HM330539.1; EU772945.1)	Not executed	Not executed
5	Kidney	Autolytic aborted fetus.	*Metamycoplasma spumans* (100% with NR_113678.1)	-	Not executed
6	Pool of tissues	Puppy from a litter from which 7 out of 11 puppies died. The dam was vaccinated against CHV-1.	*Mycoplasmopsis edwardii* (100% with LS991951.1)	-	*Escherichia coli*
7	Pool of tissues	Four puppies died at 24 h of life with hepatic lesions.	*Mycoplasmopsis canis* (100% with LR215010.1)	-	Not executed
8	Pool of tissues and vaginal swab (VS) of the bitch	Four-day-old pup.	Pool of tissues: uncultured *Mycoplasma* (100% with KM485604.1) VS: *Mycoplasmopsis edwardii* (100% with LS991951.1)	+	Not executed
9	Fetal membrane and vaginal swab (VS) of the bitch	Cesarean section due to dystocia; all puppies were healthy.	VS: repeatedly unsequentiable Fetal membrane: *Mycoplasmopsis canis* (100% with LR215010.1)	-	*Escherichia coli*, *Staphylococcus aureus*, *Proteus* sp. (probably contaminant)

* Percentage of identity and accession number of the reference microorganism.

## Data Availability

The original contributions presented in the study are included in the article, further inquiries can be directed to the corresponding author.
